# Impact of sex on outcomes after surgery for non-muscle-invasive and muscle-invasive bladder urothelial carcinoma: a systematic review and meta-analysis

**DOI:** 10.1007/s00345-022-04116-x

**Published:** 2022-08-13

**Authors:** Keiichiro Mori, Takafumi Yanagisawa, Satoshi Katayama, Ekaterina Laukhtina, Benjamin Pradere, Hadi Mostafaei, Fahad Quhal, Pawel Rajwa, Marco Moschini, Francesco Soria, David D’andrea, Mohammad Abufaraj, Simone Albisinni, Wojciech Krajewski, Wataru Fukuokaya, Jun Miki, Takahiro Kimura, Shin Egawa, Jeremy YC. Teoh, Shahrokh F. Shariat, European Association of Urology–Young Academic Urologists Urothelial Carcinoma Working Group (EAU-YAU)

**Affiliations:** 1 Department of Urology, Comprehensive Cancer Center, Medical University of Vienna, Vienna, Austria; 2 Department of Urology, The Jikei University School of Medicine, Tokyo, Japan; 3 Department of Urology, Okayama University Graduate School of Medicine, Dentistry and Pharmaceutical Sciences, Okayama, Japan; 4 Institute for Urology and Reproductive Health, Sechenov University, Moscow, Russia; 5 Research Center for Evidence Based Medicine, Tabriz University of Medical Sciences, Tabriz, Iran; 6 Department of Urology, King Fahad Specialist Hospital, Dammam, Saudi Arabia; 7 Department of Urology, Medical University of Silesia, Zabrze, Poland; 8 Department of Urology, San Raffaele Hospital, Milan, Italy; 9 Division of Urology, Department of Surgical Sciences, University of Studies of Torino, Turin, Italy; 10 Division of Urology, Department of Special Surgery, The University of Jordan, Amman, Jordan; 11 Department of Urology, University Clinics of Brussels, Hôpital Erasme, Université Libre de Bruxelles, Brussels, Belgium; 12 Department of Department of Minimally Invasive and Robotic Urology, Wrocław Medical University, Wroclaw, Poland; 13 Department of Surgery, S.H. Ho Urology Centre, The Chinese University of Hong Kong, Hong Kong, China; 14 Department of Urology, Weill Cornell Medical College, New York, NY USA; 15 Department of Urology, University of Texas Southwestern, Dallas, TX USA; 16 Karl Landsteiner Institute of Urology and Andrology, Vienna, Austria; 17 Department of Urology, Second Faculty of Medicine, Charles University, Prague, Czech Republic

**Keywords:** Sex, Meta-analysis, Muscle-invasive bladder urothelial carcinoma, Non-muscle-invasive bladder urothelial carcinoma

## Abstract

**Purpose:**

To assess the prognostic value of sex for non-muscle-invasive/muscle-invasive bladder urothelial carcinoma (NMIBC/MIBC) treated with radical surgery.

**Methods:**

The PubMed, Web of Science, and Scopus databases were searched in November 2021 according to the Preferred Reporting Items for Systematic Review and Meta-analysis statement. Studies were deemed eligible if they involved the comparison of the overall, cancer-specific, progression, and recurrence-free survival of patients with NMIBC/MIBC. Formal sex-stratified meta-analyses of these outcomes were performed.

**Results:**

Thirty-one studies, which included 32,525 patients with NMIBC, and 63 studies, which included 85,132 patients with MIBC, were eligible for review and meta-analysis. Female sex was associated with worse cancer-specific survival (pooled hazard ratio [HR], 1.21; 95% confidence interval [CI], 1.11–1.31) and overall survival (pooled HR, 1.02; 95% CI, 1.00–1.05) in patients with MIBC. In contrast, however, sex was not associated with cancer-specific survival (pooled HR, 1.01; 95% CI, 0.70–1.46), progression-free survival (pooled HR, 1.04; 95% CI, 0.88–1.24), and recurrence-free survival (pooled HR, 1.06; 95% CI, 0.98–1.16) in patients with NMIBC.

**Conclusions:**

Sex is associated with an increased risk of worse survival outcomes in patients with MIBC but not in those with NMIBC. Given the genetic and social differences between sexes, sex may represent a key factor in the clinical decision-making process.

**Supplementary Information:**

The online version contains supplementary material available at 10.1007/s00345-022-04116-x.

## Introduction

Urothelial carcinoma of the bladder (UCB) is the ninth most diagnosed cancer in the world. Approximately 75–85% of patients with UCB in developed countries present with disease confined to the mucosa or submucosa, i.e., non-muscle-invasive bladder cancer (NMIBC) [1]. Transurethral resection (TUR) is an initial clinical step in the diagnosis and management of NMIBC [2, 3]. Patients who undergo TUR generally show favorable cancer-specific survival (CSS) and overall survival (OS) [4]. However, the disease recurs in > 50% of these patients, with progression to muscle-invasive bladder cancer (MIBC) in approximately 20% of patients [5, 6]. Risk stratification of patients is key to formulating an appropriate management strategy in light of their probability of recurrence and progression [7]. Radical cystectomy (RC) with lymph node dissection remains the standard treatment for very high-risk NMIBC and MIBC [1, 8]. Despite definitive therapy with curative intent, the 5-year OS of patients remains poor (< 60%) [9, 10]. Thus, various clinical and pathological factors have been explored to improve the risk stratification of patients with UCB to facilitate clinical decision-making and patient counseling [11–16].

Sex-specific differences in the outcomes of UCB have been extensively investigated [14]. Men have a threefold greater risk of developing UCB than women. Additionally, although the incidence of UCB increased 25% faster in men than in women over the past decade, the female sex was also found to be an independent adverse prognostic factor for UCB [17, 18]. Smoking habits, tumor biology, occupational risk factors, response to BCG therapy/immunotherapy, and anatomical and hormonal factors may affect these reported sex-specific disparities in UCB statistics [14]**.** However, no meta-analysis on the sex-stratified comparison of the outcomes of NMIBC and MIBC has been conducted to date. Moreover, sex-specific differences in the outcomes of UCB are often overlooked in the clinical practice. Understanding the factors associated with disparities in the presentation, evaluation, and management of UCB in men and women could result in improvements in the timeliness and intensity of healthcare delivery [19]. For instance, it should be deemed crucial to ensure that women with suspected hematuria likely due to infection they are prone to, e.g., cystitis, are screened for UCB as well, just as men are, to avoid overlooking it, given that reliable detection of early-stage UCB in women is likely to lead to improvements in their life prognosis. In addition, such efforts may lessen the magnitude of the sex-specific disparities in UCB outcomes [19]. Therefore, the aim of this systematic review and meta-analysis was to summarize the existing data and determine whether sex-specific differences may predict oncological outcomes in patients treated with surgery for NMIBC and MIBC. This study also explored the effects of sex on the prognosis of UCB.

## Methods

### Search strategy

The systematic review and meta-analysis were performed according to the Preferred Reporting Items for Systematic Reviews and Meta-analyses (PRISMA) statement [20]. The PubMed, Web of Science, and Scopus databases were searched in November 2021 to identify reports on the prognostic value of sex in UCB. The keywords used in our search strategy were: (transurethral resection OR cystectomy) AND (gender OR sex) AND (survival OR mortality). The primary outcomes of interest in NMIBC were recurrence-free survival (RFS) and progression-free survival (PFS) with the secondary outcomes being CSS. The primary outcome of interest in MIBC was CSS with the secondary outcomes being OS. Initial screening was performed independently by two investigators based on titles and abstracts to exclude ineligible reports, and the reasons for exclusions were noted. Potentially relevant reports were subjected to a full-text review and their relevance confirmed after the data extraction process. Disagreements were resolved via consensus with a third investigator.

### Inclusion and exclusion criteria

Studies were included if they had involved female patients treated for UCB (Patients) who had received surgery (Intervention) compared to male patients (Comparison) to assess the independent predictive value of sex on CSS, OS, RFS, and PFS (Outcome) utilizing multivariate Cox regression analysis (Study design) in nonrandomized observational, randomized, or cohort studies. Excluded from analysis were reviews, letters, editorials, meeting abstracts, replies from authors, case reports and non-English articles. Where the database studies were concerned, our analysis was limited to one national database study to avoid redundancies. In case of duplicate publications on the same study population, the study of a higher quality or of the most recent date was selected. The references listed in the manuscripts included were also explored for further studies of interest.

### Data extraction

Two investigators independently extracted the following information from the included articles: first author’s name, publication year, country of recruitment, period of patient recruitment, number of patients, age, sex, oncological outcome, and follow-up duration. Subsequently, hazard ratios (HR) and 95% confidence intervals (CI) were extracted from multivariate analyses for any sex difference found to be associated with each outcome of interest and all discrepancies in data extraction were resolved by consensus with a third investigator.

### Quality assessment

The Newcastle–Ottawa Scale (NOS) was used to assess the quality of the nonrandomized studies included according to the Cochrane Handbook for Systematic Reviews of Interventions. The scale rates selection (1–4 points), comparability (1–2 points), and exposure (1–3 points), with total scores ranging from 0 (lowest) to 9 (highest). The main confounders were identified as the important prognostic factors of cancer-specific, overall, progression-free, and recurrence-free survival. The presence of confounders was determined by consensus and review of the literature. We identified studies with scores above 6 as high-quality choices.

### Risk of bias (RoB) assessment

Each of the studies included in this analysis was assessed for RoB according to the Cochrane Handbook for Systematic Reviews of Interventions (Supplementary Fig. 1, Supplementary Fig. 2). Due to the nature of these nonrandomized studies, the RoB was determined for each study by examining the risk of pre-assigned confounders. The confounding factors were identified as the most important of prognostic factors at the time of treatment. Each study was assessed for RoB independently by two authors, with the overall RoB level defined as “low”, “intermediate” or “high”.

### Statistical analyses

Forest plots were used to assess and summarize the multivariate HRs to describe the relationships between sex differences and CSS, OS, RFS, and PFS. Studies were not considered eligible for meta-analysis if they had used Kaplan–Meier log-rank, univariate Cox proportional hazard regression, or general logistic regression analyses. For all studies reporting only HRs and *P* values, the corresponding 95% CIs were calculated [21, 22]. The studies included in the meta-analysis were evaluated for heterogeneity in outcome using the Cochrane’s *Q* test and the *I*^2^ statistic. Significant heterogeneity was indicated by a *P* < 0.05 in Cochrane’s *Q* tests and a ratio of > 50% in *I*^2^ statistics. Fixed-effects models were used to calculate pooled HRs for non-heterogeneous outcomes [23–25]. Sensitivity analyses were conducted to assess the robustness of the results based on the quality of the studies included. All statistical analyses were performed using Review Manager 5.3 and Stata/MP 14.2 (Stata Corp., College Station, TX) with the level of statistical significance set at *P* < 0.05.

## Results

### Study selection and characteristics

Our initial search identified a total of 1512 publications; of these, a total of 1243 were available after exclusion of duplicates (Supplementary Fig. 3). A total of 1,071 articles were excluded after screening of their titles and abstracts, and 172 articles were available for full-text review. A total of 31 studies in NMIBC which accounted for 32,525 patients, as well as 63 studies in MIBC which accounted for 85,132 patients, were identified as meeting the selection criteria for the current meta-analysis. Data extracted from the 94 studies are summarized in Supplementary Tables 1 and 2. All studies included in this study were published between 2003 and 2021 with 31, 21, 23, and 18 conducted in Europe, North America, Asia and internationally, and with patient accrual for these studies occurring between 1971 and 2020. Males and females accounted for 25,317 (77.8%) and 7208 (22.2%), respectively, in the NMIBC cohort, as well as for 65,986 (77.5%) and 19,146 (22.5%), respectively, in the MIBC cohort (median age, 61.4–75 years), with a median follow-up of 10.2–223.2 months.

### Meta-analysis

#### Association of sex difference with CSS in MIBC

**Fig. 1:**
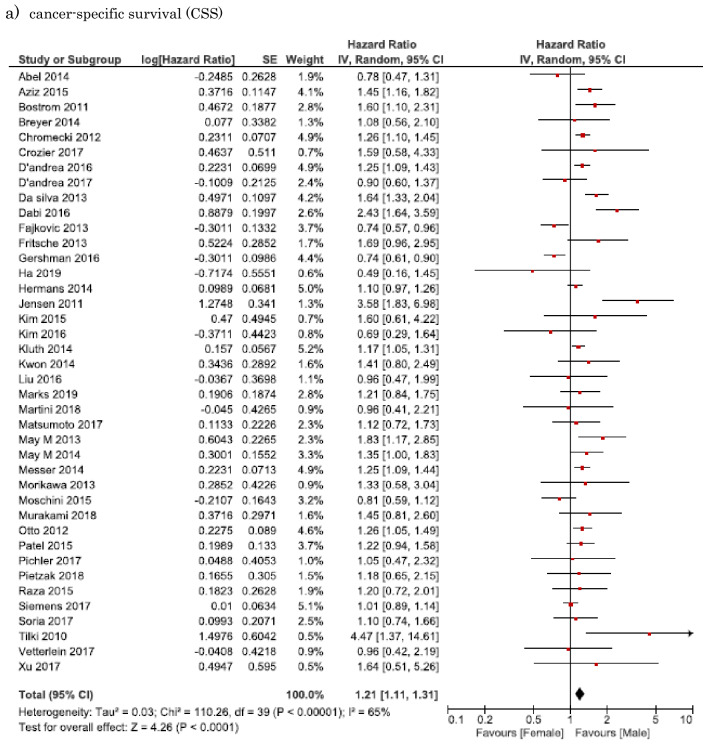

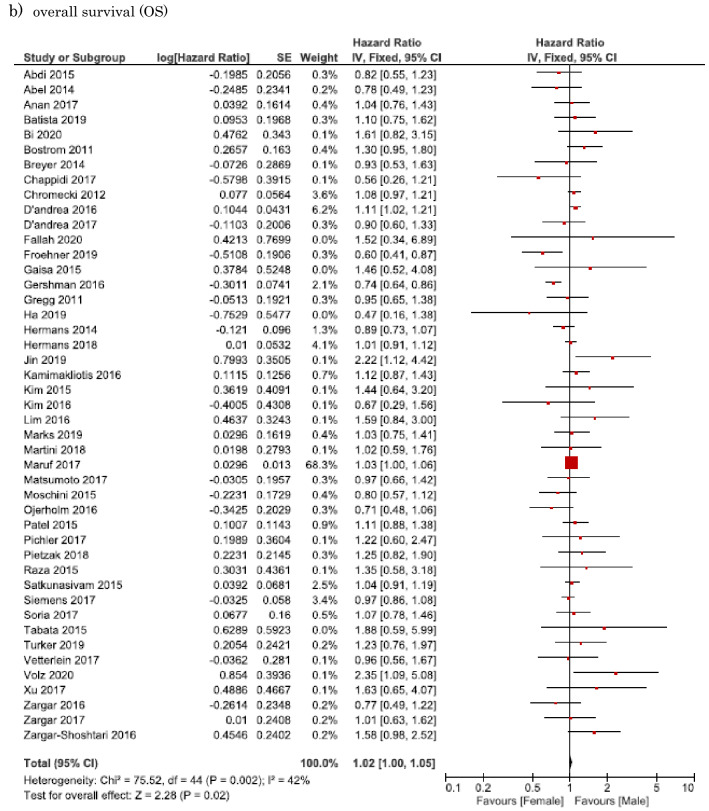
Forest plots showing the association between sex and oncological outcomes of muscle-invasive bladder urothelial carcinoma. **a** Cancer-specific survival (CSS); **b** overall survival (OS)

Forty-two studies involving 42,794 patients provided data on the association of sex difference with CSS in MIBC. The forest plot (Fig. 1A) revealed that sex difference was significantly associated with CSS in MIBC (pooled HR, 1.21; 95% CI, 1.11–1.31). The Cochrane’s *Q* test (*P* < 0.001) and *I*^2^ test (*I*^2^ = 65%) revealed significant heterogeneity.

#### Association of sex difference with OS in MIBC

Forty-five studies involving 63,170 patients provided data on the association of sex difference with OS in MIBC. The forest plot (Fig. 1B) revealed that sex difference was significantly associated with OS in MIBC (pooled HR, 1.02; 95% CI, 1.00–1.05). The Cochrane’s *Q* test (*P* = 0.002) and *I*^2^ test (*I*^2^ = 42%) revealed no significant heterogeneity.

#### Association of sex difference with PFS in NMIBC

**Fig. 2:**
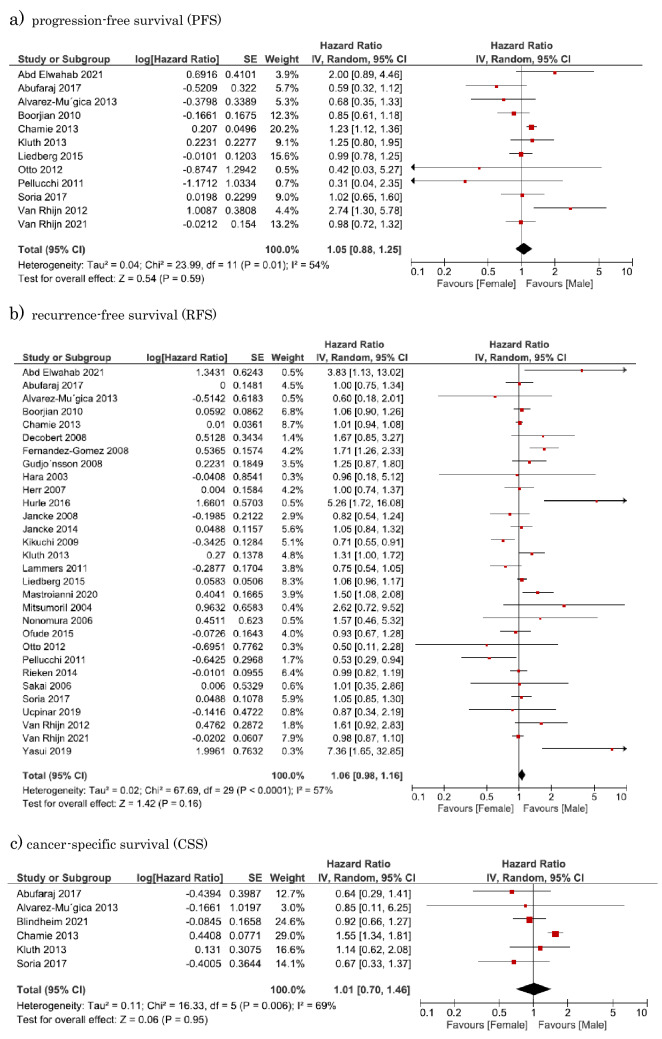
Forest plots showing the association between sex and oncological outcomes of non-muscle-invasive bladder urothelial carcinoma. **a** Progression-free survival (PFS); **b** recurrence-free survival (RFS); c) cancer-specific survival (CSS)

Twelve studies involving 22,934 patients provided data on the association of sex difference with PFS in NMIBC. The forest plot (Fig. 2A) revealed that sex difference was not significantly associated with PFS in NMIBC (pooled HR, 1.04; 95% CI, 0.88–1.24). The Cochrane’s *Q* test (*P* = 0.016) and *I*^2^ test (*I*^2^ = 52.7%) revealed significant heterogeneity.

#### Association of sex difference with RFS in NMIBC

Thirty studies involving 31,408 patients provided data on the association of sex difference with RFS in NMIBC. The forest plot (Fig. 2B) revealed that sex difference was not significantly associated with RFS in NMIBC (pooled HR, 1.06; 95% CI, 0.98–1.16). The Cochrane’s *Q* test (*P* ≤ 0.001) and *I*^2^ test (*I*^2^ = 57.2%) revealed significant heterogeneity.

#### Association of sex difference with CSS in NMIBC

Six studies involving 11,026 patients provided data on the association of sex difference with CSS in NMIBC. The forest plot (Fig. 2C) revealed that sex difference was not significantly associated with RFS in NMIBC (pooled HR, 1.01; 95% CI, 0.70–1.46). The Cochrane’s *Q* test (*P* = 0.006) and *I*^2^ test (*I*^2^ = 69%) revealed significant heterogeneity.

### Sensitivity analyses

Sensitivity analyses were performed based on the quality of the studies included, which demonstrated that the overall HRs were not significantly influenced in high-quality group, suggesting the robustness and reliability of the results in our meta-analysis.

## Discussion

In this systematic review and meta-analysis, we investigated the prognostic value of sex for UCB. The results showed that female patients with MIBC have a significantly increased risk for worse CSS and OS compared to their male counterparts. However, there were no sex-associated differences in the prognosis of patients with NMIBC.

Our findings on the outcomes of MIBC corroborate the frequently reported fact that female patients with UCB are at a prognostically and clinically significant risk of succumbing to their disease. There are multiple reasons for this disparity. First, women with UCB are more likely to present with advanced cancer and are often older, which is a risk factor for poorer outcomes after RC [26, 27]. Second, hematuria and irritative voiding symptoms in female patients are first interpreted as symptoms of gynecologic diseases or urinary tract infections, leading to delays in the detection of UCB in women [28, 29]. Delayed UCB diagnosis increases cancer mortality, independent of the cancer stage and grade [30]. Thus, continued efforts are warranted to educate physicians on the need to follow a standardized diagnostic approach for patients with hematuria, regardless of sex [31, 32]. Third, it is hypothesized that embryologic and anatomical differences between men and women account for the sex-specific differences in UCB prognoses. The embryonic development of the trigone and the posterior bladder neck, which form a common origin with the upper part of the vagina, may contribute to the more invasive extension of UCB in women [33]. In contrast to men whose prostate and Denonvilliers’ fascia prevent direct tumor spread and lymphovascular invasion from the bladder neck to adjacent organs, women have no barriers in place between the bladder neck and the anterior vaginal wall [34]. Thus, the prognosis of UCB varies widely between sexes, as observed in pT4 disease involving vaginal or prostate invasion. Another relevant anatomic difference between men and women is bladder wall thickness. With advancing age, men develop thicker detrusor muscles secondary to changes caused by the resistance associated with benign prostatic hyperplasia [35]. While these anatomical differences between the sexes may affect UCB prognosis, it has been shown that the impact of these differences on prognosis is particularly significant in MIBC, but limited in NMIBC. In other words, if the disease remains in the NMIBC stage in women, it is likely to be diagnosed relatively earlier, thus causing minimal anatomical damage and reducing the magnitude of the differences between the prognosis of UCB in men and women. However, this is only a theoretical hypothesis that is yet to be validated. Indeed, the results of the present meta-analysis did not show any significant differences in RFS, PFS, and CSS between men and women with NMIBC.

Another hypothesis regarding the prognostic differences between male and female patients with UCB is the influence of sex steroids and their receptors. Numerous studies have indicated a potential role of androgens and estrogens and their receptors in influencing the development and course of UCB [36, 37]. For example, the expression of the androgen receptor (AR) is inversely correlated with UCB stage, suggesting that its expression may promote carcinogenesis [38, 39]. Loss of AR responsiveness may account for an increased risk of disease progression via an androgen-independent mechanism, a hypothesis that could explain the earlier progression of UCB in women [40, 41]. In contrast, female sex hormones, predominantly estrogens, protect against bladder tumorigenesis through the estrogen receptor (ER) pathway [42]. ER expression in the female bladder is not surprising, given the common embryological origin of the bladder trigone and the upper portion of the vagina. Furthermore, several lines of epidemiological evidence suggest a reduced risk for UCB in women who had menarche at an older age, those with multiparity, and those receiving combined estrogen and progestin hormone replacement therapy [43–45], suggesting a role of sex steroids in the risk for UCB. However, the exact effect of sex steroids and their receptors on UCB is not fully established yet.

Sex-specific differences have been noted in the responses to various therapeutic agents. Sex-specific differences in immunity remain a critical factor that dictates host immune responses [46, 47]. Notably, women are more likely to be affected by urinary tract infection or painful bladder syndrome than men, suggesting sex-specific differences in immunity in the bladder. In the context of infection, which may be analogous to BCG immunotherapy, there are stark differences in the innate immune responses of men and women, given the presence of non-commensal bacteria in the bladder lumen [48]. Increased susceptibility to urinary tract infections among postmenopausal women is also attributed to their lower estrogen levels. These sex-specific disparities may be attributed to the influence of estrogen signaling on BCG response [49]. In BCG response, the antitumor properties of BCG are mediated by the binding of BCG to bladder cells via the integrin-a5b1 receptor complex, which is upregulated by the cytokine interleukin (IL)-6 [50], whose expression is, in turn, inhibited by estrogen [51]. Furthermore, blocking the binding of BCG to the bladder mucosa has been shown to inhibit BCG-related antitumor activity [52], which was recently reported to occur due to pathway downregulation [49]. In the context of PD-1/PD-L1 targeting immunotherapy, sex has recently been reported as an important variable in determining response to treatment, which explains the poor response in women [53, 54]. Notably, the expression of PD-L1 is also regulated by estrogen and several X-linked micro-RNAs [55]. Along with these regulatory mechanisms, the increased expression of PD-L1 and/or other immune checkpoints is likely implicated in the female bladder, which is exposed to more pathogenic microbial challenges, as well as higher estrogen levels, than the male bladder [54]. However, further studies of different cancer states that are conducted under normal physiological conditions are warranted to obtain definitive supporting evidence of the clinical utility of this hypothesis.

Another available line of evidence is that smoking, the most established risk factor for UCB, has a larger impact in women than in men [56, 57]. Although smoking rates are generally declining, tobacco use has recently increased among women and is expected to double between 2005 and 2025. In addition, although cigarette consumption is still estimated to be higher in men than in women, female smokers are not only at greater risk of developing UCB [58], but also have worse prognoses than male smokers [59].

Despite the common assertion that the urinary tract is sterile, evidence of the presence of a commensal bacterial community in the urinary tract emerged as early as 1997 [48]. Notably, greater diversity was observed between healthy women and those with bacterial vaginosis than between male and female microbiota samples. Although reports of differences between the microbiome of patients with UCB and those of healthy individuals are beginning to emerge, no study has been conducted to directly assess how the urinary microbiome may change during tumorigenesis, intravesical instillation of therapeutic agents, or systemic administration of anticancer treatments, or to determine whether there may be changes or differences between the sexes in any of these aspects [60]. In addition to appropriate powering of any future study, careful planning of sample acquisition, storage, and analysis are important for an accurate depiction of the urinary microbiome.

This study had several limitations. First, all the studies included in the analysis were retrospective studies; thus, the risk of selection bias in the analysis is increased. Second, reporting bias may have led to negative results not being published. Third, heterogeneity was detected in the analyses, suggesting the limited value of these results. As a result, these papers were not of equal rank (The weight in analyses is far difference among each studies). Moreover, the study did not adequately address the heterogeneity in methods for determining survival (local databases or social security/national registries) or progression/recurrence (radiographic or biopsy-proven). Although a random-effects model was used to minimize heterogeneity among the studies, the conclusions should be interpreted with caution. Fourth, the chemotherapy protocols used in the included studies were heterogeneous; hence, the analysis of individual treatment strategies was limited. Moreover, our analysis was not adjusted for known sex-specific differences in response to immunotherapy, including BCG. Furthermore, as no studies were excluded from analysis in this study on account of the operative methods (e.g., lymph node dissection and urinary diversion) involved, which varied between the studies. Therefore, the possibility cannot be ruled out that this may have affected our study results and contributed to heterogeneity among the studies evaluated, and our results need to be interpreted with these factors in mind. Fifth, of the patients evaluated, some were found to have progressed from NMIBC to MIBC, likely suggesting an overlap of the two populations. Again, those with prior NMIBC and those with MIBC should have been evaluated separately in the first place; however, the data available from the studies included in this analysis did not allow for this analysis, which constituted a limitation of our study. Sixth, high-risk NMIBC patients were not excluded from MIBC patients. Thus, it must be borne in mind that those undergoing RC included not only MIBC but high-risk NMIBC patients. Seventh, while sex difference was significantly associated with OS in MIBC, the HR included 1.0 and was subject to rounding. Finally, automation was not used to screen the initial 1,512 articles made available prior to independent investigator reviews. Therefore, well-designed, prospective studies with long follow-up periods are needed to validate the prognostic value of sex-specific differences for UCB in the clinical setting, as well as to determine whether consideration of these differences may improve the current risk stratification tools and clinical decision-making for patients with UCB.

## Conclusion

Current evidence suggests that the female sex is a negative prognostic factor for survival after radical surgery for MIBC. In contrast, sex was not associated with adverse survival outcomes in patients with NMIBC. Further study is warranted to verify whether sex is a potential prognostic factor worth including in validated prognostic tables and nomograms to facilitate more accurate diagnosis and risk stratification of patients with UCB.

## Supplementary Information

Below is the link to the electronic supplementary material.

Supplementary file1 (DOCX 90 KB)

Supplementary file2 (DOCX 58 KB)

Supplementary file3 (DOCX 23 KB)

Supplementary file4 (DOCX 18 KB)

Supplementary file5 (DOCX 19 KB)

Supplementary file6 (DOCX 24 KB)
