# Supplementary material for: Impact of sex on outcomes after surgery for non-muscle-invasive and muscle-invasive bladder urothelial carcinoma: a systematic review and meta-analysis
Source: World J Urol. 2022 Aug 13;41(4):909–19. doi: 10.1007/s00345-022-04116-x (PMC10159976; doi:10.1007/s00345-022-04116-x)
Supplement: Supplementary file 1 — Supplementary file1 (DOCX 90 KB) [file 345_2022_4116_MOESM1_ESM.docx]

**Supplementary Figure 1**

Risk　of　bias　summary　of　the　studies　that analyses the　association　between　sex　difference and　risk　of　disease　mortality　in　 muscle-invasive bladder cancer

| **Author, year** | **A** | B | C | D | E | F | G | H | I | J | K | L | M | N |
| --- | --- | --- | --- | --- | --- | --- | --- | --- | --- | --- | --- | --- | --- | --- |
| **Tilki 2010** | 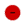 | 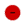 | 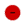 | 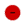 | 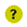 | 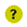 | 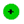 | 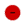 | 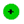 | 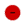 | 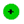 | 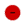 | 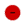 | 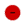 |
| **Bostrom 2011** | 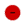 | 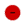 | 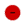 | 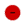 | 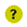 | 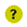 | 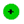 | 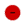 | 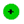 | 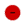 | 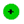 | 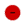 | 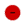 | 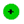 |
| **Gregg 2011** | 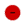 | 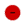 | 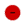 | 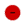 | 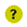 | 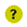 | 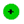 | 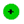 | 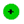 | 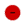 | 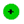 | 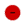 | 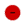 | 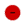 |
| **Jensen 2011** | 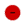 | 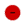 | 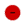 | 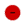 | 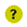 | 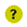 | 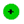 | 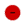 | 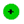 | 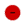 | 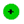 | 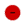 | 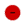 | 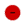 |
| **Choromecki 2012** | 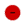 | 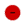 | 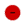 | 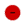 | 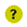 | 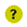 | 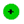 | 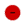 | 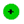 | 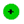 | 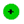 | 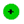 | 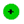 | 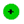 |
| **Otto2012** | 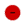 | 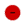 | 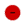 | 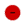 | 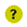 | 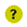 | 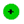 | 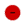 | 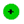 | 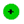 | 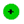 | 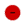 | 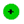 | 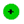 |
| **Da silva 2013** | 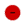 | 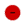 | 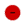 | 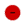 | 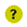 | 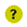 | 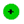 | 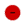 | 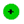 | 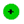 | 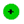 | 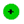 | 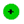 | 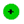 |
| **Fajkovic 2013** | 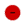 | 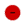 |  |  |  |  |  |  |  |  |  |  |  |  |
| **Fritsche 2013** |  |  |  |  |  |  |  |  |  |  |  |  |  |  |
| **May M 2013** |  |  |  |  |  |  |  |  |  |  |  |  |  |  |
| **Morikawa 2013** |  |  |  |  |  |  |  |  |  |  |  |  |  |  |
| **Abel 2014** |  |  |  |  |  |  |  |  |  |  |  |  |  |  |
| **Breyer 2014** |  |  |  |  |  |  |  |  |  |  |  |  |  |  |
| **Hermans T 2014** |  |  |  |  |  |  |  |  |  |  |  |  |  |  |
| **Kluth 2014** |  |  |  |  |  |  |  |  |  |  |  |  |  |  |
| **Kwon 2014** |  |  |  |  |  |  |  |  |  |  |  |  |  |  |
| **May M 2014** |  |  |  |  |  |  |  |  |  |  |  |  |  |  |
| **Messer 2014** |  |  |  |  |  |  |  |  |  |  |  |  |  |  |
| **Abdi 2015** |  |  |  |  |  |  |  |  |  |  |  |  |  |  |
| **Aziz 2015** |  |  |  |  |  |  |  |  |  |  |  |  |  |  |
| **Gaisa 2015** |  |  |  |  |  |  |  |  |  |  |  |  |  |  |
| **Kim HS2015** |  |  |  |  |  |  |  |  |  |  |  |  |  |  |
| **Moschini 2015** |  |  |  |  |  |  |  |  |  |  |  |  |  |  |
| **Patel 2015** |  |  |  |  |  |  |  |  |  |  |  |  |  |  |
| **Raza 2015** |  |  |  |  |  |  |  |  |  |  |  |  |  |  |
| **Satkunasivam 2015** |  |  |  |  |  |  |  |  |  |  |  |  |  |  |
| **Tabata 2015** |  |  |  |  |  |  |  |  |  |  |  |  |  |  |
| **Dabi 2016** |  |  |  |  |  |  |  |  |  |  |  |  |  |  |
| **D'andrea 2016** |  |  |  |  |  |  |  |  |  |  |  |  |  |  |
| **Gershman 2016** |  |  |  |  |  |  |  |  |  |  |  |  |  |  |
| **Kamimakliotis 2016** |  |  |  |  |  |  |  |  |  |  |  |  |  |  |
| **Kim TH 2016** |  |  |  |  |  |  |  |  |  |  |  |  |  |  |
| **Lim 2016** |  |  |  |  |  |  |  |  |  |  |  |  |  |  |
| **Liu 2016** |  |  |  |  |  |  |  |  |  |  |  |  |  |  |
| **Ojerholm 2016** |  |  |  |  |  |  |  |  |  |  |  |  |  |  |
| **Zargar 2016** |  |  |  |  |  |  |  |  |  |  |  |  |  |  |
| **Zargar-Shoshtari 2016** |  |  |  |  |  |  |  |  |  |  |  |  |  |  |
| **Anan 2017** |  |  |  |  |  |  |  |  |  |  |  |  |  |  |
| **Chappidi 2017** |  |  |  |  |  |  |  |  |  |  |  |  |  |  |
| **Crozier 2017** |  |  |  |  |  |  |  |  |  |  |  |  |  |  |
| **D'andrea 2017** |  |  |  |  |  |  |  |  |  |  |  |  |  |  |
| **Maruf 2017** |  |  |  |  |  |  |  |  |  |  |  |  |  |  |
| **Matsumoto 2017** |  |  |  |  |  |  |  |  |  |  |  |  |  |  |
| **Pichler 2017** |  |  |  |  |  |  |  |  |  |  |  |  |  |  |
| **Siemens 2017** |  |  |  |  |  |  |  |  |  |  |  |  |  |  |
| **Soria 2017** |  |  |  |  |  |  |  |  |  |  |  |  |  |  |
| **Vetterlein 2017** |  |  |  |  |  |  |  |  |  |  |  |  |  |  |
| **Xu 2017** |  |  |  |  |  |  |  |  |  |  |  |  |  |  |
| **Zargar 2017** |  |  |  |  |  |  |  |  |  |  |  |  |  |  |
| **Hermans TJN 2018** |  |  |  |  |  |  |  |  |  |  |  |  |  |  |
| **Martini T 2018** |  |  |  |  |  |  |  |  |  |  |  |  |  |  |
| **Murakami 2018** |  |  |  |  |  |  |  |  |  |  |  |  |  |  |
| **Pietzak 2018** |  |  |  |  |  |  |  |  |  |  |  |  |  |  |
| **Batista 2019** |  |  |  |  |  |  |  |  |  |  |  |  |  |  |
| **Bi 2019** |  |  |  |  |  |  |  |  |  |  |  |  |  |  |
| **Froehner 2019** |  |  |  |  |  |  |  |  |  |  |  |  |  |  |
| **Ha 2019** |  |  |  |  |  |  |  |  |  |  |  |  |  |  |
| **Jin 2019** |  |  |  |  |  |  |  |  |  |  |  |  |  |  |
| **Marks 2019** |  |  |  |  |  |  |  |  |  |  |  |  |  |  |
| **Martini A 2019** |  |  |  |  |  |  |  |  |  |  |  |  |  |  |
| **Turker 2019** |  |  |  |  |  |  |  |  |  |  |  |  |  |  |
| **Fallah 2020** |  |  |  |  |  |  |  |  |  |  |  |  |  |  |
| **Volz 2020** |  |  |  |  |  |  |  |  |  |  |  |  |  |  |

(A) Random sequence generation (selection bias); (B) allocation concealment (selection bias); (C) blinding of outcome assessment (detection bias); (D) blinding of outcome assessment (detection bias); (E) incomplete outcome data (attrition bias); (F) selective reporting (reporting bias); and adjustment for the effects of the following confounders: age (G), variant histology (H), pT stage (I), pathologic grade (J), pN stage (K), positive surgical margins (L), lymphovascular invasion (M) and perioperative chemotherapy (N). Green circles represent a low risk of bias and confounding, red circles represent a high risk of bias and confounding, and yellow circles represent an unclear risk of bias and confounding.
