# Supplementary material for: Impact of sex on outcomes after surgery for non-muscle-invasive and muscle-invasive bladder urothelial carcinoma: a systematic review and meta-analysis
Source: World J Urol. 2022 Aug 13;41(4):909–19. doi: 10.1007/s00345-022-04116-x (PMC10159976; doi:10.1007/s00345-022-04116-x)
Supplement: Supplementary file 2 — Supplementary file2 (DOCX 58 KB) [file 345_2022_4116_MOESM2_ESM.docx]

**Supplementary Figure 2**

Risk　of　bias　summary　of　the　studies　that analyses the　association　between　sex　difference and　risk　of　disease　recurrence, progression, and mortality　in　non-muscle-invasive bladder carcinoma

| **Author, year** | **A** | B | C | D | E | F | G | H | I | J | K | L | M | N |
| --- | --- | --- | --- | --- | --- | --- | --- | --- | --- | --- | --- | --- | --- | --- |
| Hara 2003 | 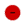 | 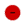 | 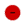 | 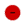 | 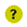 | 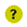 | 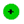 | 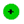 | 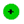 | 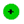 | 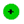 | 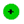 | 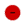 | 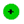 |
| Mitsumori 2004 | 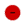 | 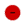 | 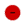 | 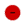 | 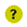 | 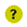 | 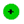 | 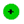 | 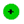 | 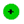 | 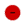 | 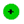 | 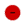 | 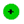 |
| Nonomura 2006 | 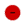 | 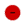 | 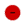 | 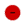 | 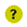 | 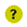 | 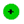 | 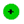 | 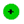 | 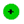 | 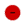 | 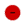 | 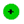 | 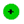 |
| Sakai 2006 | 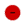 | 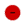 | 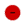 | 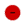 | 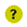 | 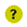 | 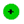 | 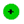 | 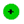 | 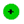 | 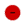 | 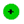 | 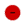 | 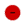 |
| Herr 2007 | 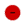 | 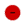 | 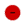 | 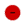 | 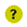 | 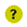 | 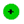 | 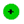 | 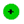 | 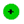 | 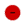 | 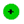 | 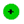 | 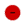 |
| Decobert 2008 | 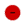 | 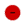 | 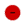 | 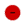 | 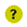 | 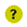 | 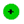 | 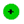 | 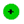 | 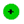 | 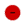 | 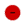 | 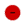 | 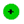 |
| Fernandez 2008 | 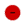 | 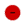 | 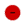 | 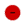 | 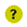 | 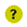 | 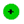 | 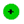 | 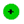 | 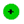 | 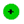 | 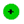 | 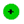 | 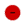 |
| Gudjonsson 2008 | 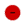 | 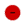 |  |  |  |  |  |  |  |  |  |  |  |  |
| Jancke 2008 |  |  |  |  |  |  |  |  |  |  |  |  |  |  |
| Kikuchi 2009 |  |  |  |  |  |  |  |  |  |  |  |  |  |  |
| Boojian 2010 |  |  |  |  |  |  |  |  |  |  |  |  |  |  |
| Lammers 2011 |  |  |  |  |  |  |  |  |  |  |  |  |  |  |
| Pellucchi 2011 |  |  |  |  |  |  |  |  |  |  |  |  |  |  |
| Otto 2012 |  |  |  |  |  |  |  |  |  |  |  |  |  |  |
| van Rhijn 2012 |  |  |  |  |  |  |  |  |  |  |  |  |  |  |
| Alvarez 2013 |  |  |  |  |  |  |  |  |  |  |  |  |  |  |
| Chamie 2013 |  |  |  |  |  |  |  |  |  |  |  |  |  |  |
| Kluth 2013 |  |  |  |  |  |  |  |  |  |  |  |  |  |  |
| Jancke 2014 |  |  |  |  |  |  |  |  |  |  |  |  |  |  |
| Rieken 2014 |  |  |  |  |  |  |  |  |  |  |  |  |  |  |
| Liedberg 2015 |  |  |  |  |  |  |  |  |  |  |  |  |  |  |
| Ofude 2015 |  |  |  |  |  |  |  |  |  |  |  |  |  |  |
| Hurle 2016 |  |  |  |  |  |  |  |  |  |  |  |  |  |  |
| Abufaraj 2017 |  |  |  |  |  |  |  |  |  |  |  |  |  |  |
| Soria 2017 |  |  |  |  |  |  |  |  |  |  |  |  |  |  |
| Ucpinar 2019 |  |  |  |  |  |  |  |  |  |  |  |  |  |  |
| Yasui 2019 |  |  |  |  |  |  |  |  |  |  |  |  |  |  |
| Mastroianni 2020 |  |  |  |  |  |  |  |  |  |  |  |  |  |  |
| Abd Elwahab 2021 |  |  |  |  |  |  |  |  |  |  |  |  |  |  |
| Blindheim 2021 |  |  |  |  |  |  |  |  |  |  |  |  |  |  |
| van Rhijn 2021 |  |  |  |  |  |  |  |  |  |  |  |  |  |  |

(A) Random sequence generation (selection bias); (B) allocation concealment (selection bias); (C) blinding of outcome assessment (detection bias); (D) blinding of outcome assessment (detection bias); (E) incomplete outcome data (attrition bias); (F) selective reporting (reporting bias); and adjustment for the effects of the following confounders: age (G), multiplicity (H), T stage (I), grade (J), CIS (K), tumor size (L), number of recurrence (M) and intra-vesical therapy (N). Green circles represent a low risk of bias and confounding, red circles represent a high risk of bias and confounding, and yellow circles represent an unclear risk of bias and confounding.
