# Supplementary material for: Impact of sex on outcomes after surgery for non-muscle-invasive and muscle-invasive bladder urothelial carcinoma: a systematic review and meta-analysis
Source: World J Urol. 2022 Aug 13;41(4):909–19. doi: 10.1007/s00345-022-04116-x (PMC10159976; doi:10.1007/s00345-022-04116-x)
Supplement: Supplementary file 3 — Supplementary file3 (DOCX 23 KB) [file 345_2022_4116_MOESM3_ESM.docx]

Supplementary Table 1 Study Characteristics (muscle invasive bladder cancer)

| Author | Year | N | Recruitment | Outcome | Region | Age | Sex (M/F) | follow up (M) | NOS |
| --- | --- | --- | --- | --- | --- | --- | --- | --- | --- |
| Tilki | 2010 | 228 | 1979-2008 | CSS | International | 66.1 | 188/40 | 48.2 | 7 |
| Bostrom | 2011 | 546 | 1986-2008 | CSS,OS | International | NR | 427/119 | 50 | 7 |
| Gregg | 2011 | 538 | 2000-2008 | OS | North America | 68.3 | 420/115 | 31.3 | 7 |
| Jensen | 2011 | 167 | 2004-2009 | CSS | Europe | 63 | 126/41 | 53 | 7 |
| Chromecki | 2012 | 4118 | 1979-2008 | CSS,OS | International | 67 | 3279/839 | 44 | 7 |
| Otto | 2012 | 2483 | 1989-2008 | CSS | Europe | 66.4 | 1976/507 | 42 | 7 |
| Da silva | 2013 | 1502 | 1992-2008 | CSS | International | 65.5 | 1177/325 | 34 | 7 |
| Fajkovic | 2013 | 748 | 1979-2008 | CSS | International | 66.9 | 609/139 | 27 | 7 |
| Fritsche | 2013 | 158 | 2006-2010 | CSS | Europe | 69 | 121/37 | 20 | 6 |
| May M | 2013 | 228 | 1989-2009 | CSS | Europe | 65.9 | 192/36 | 30 | 7 |
| Morikawa | 2013 | 82 | 1990-2005 | CSS | Asia | NR | 68/14 | 68 | 7 |
| Abel | 2014 | 360 | 2002-2012 | CSS,OS | North America | 67.9 | 284/76 | 18.7 | 6 |
| Breyer | 2014 | 116 | 1989-2010 | CSS,OS | Europe | 73 | 87/29 | 15 | 6 |
| Hermans T | 2014 | 424 | 1992-2012 | CSS,OS | North America | 70.1 | 325/99 | 58.4 | 7 |
| Kluth | 2014 | 8102 | 1971-2012 | CSS | International | NR | 6497/1605 | NR | 7 |
| Kwon | 2014 | 746 | 1990-2012 | CSS | Asia | 62.4 | 664/82 | 64.3 | 7 |
| May M | 2014 | 521 | 1992-2007 | CSS | Europe | 65.2 | 388/133 | 65 | 7 |
| Messer | 2014 | 4216 | 1979-2008 | CSS | North America | NR | 3326/890 | 31.5 | 7 |
| Abdi | 2015 | 314 | 2008-2013 | OS | North America | NR | 252/62 | NR | 6 |
| Aziz | 2015 | 856 | 1989-2011 | CSS | International | 68 | 714/142 | 40 | 7 |
| Gaisa | 2015 | 121 | 2003-2009 | OS | Europe | 68.5 | 84/37 | 21.5 | 6 |
| Kim HS | 2015 | 101 | 1991-2012 | CSS,OS | Asia | 69 | 86/15 | 32.5 | 7 |
| Moschini | 2015 | 1490 | 1990-2013 | CSS,OS | Europe | 68 | 1245/245 | 110 | 7 |
| Patel | 2015 | 804 | 2001-2009 | CSS,OS | Asia | NR | 610/194 | 62.5 | 7 |
| Raza | 2015 | 702 | 2003- | CSS,OS | International | 69 | 569/133 | 67 | 7 |
| Satkunasivam | 2015 | 2047 | 1971-2009 | OS | North America | NR | 1621/426 | 148.8 | 7 |
| Tabata | 2015 | 65 | 1990-2013 | OS | Asia | 66 | 54/11 | 26.1 | 7 |
| Dabi | 2016 | 701 | 1995-2011 | CSS | Europe | NR | 553/148 | 45 | 7 |
| D'andrea | 2016 | 4198 | 1990-2012 | CSS,OS | International | 67 | 3362/836 | 42.4 | 7 |
| Gershman | 2016 | 2086 | 1980-2008 | CSS,OS | North America | 68 | 1712/374 | 132 | 7 |
| Kamimakliotis | 2016 | 919 | 1990-2013 | OS | North America | 66.2 | 727/192 | 77 | 7 |
| Kim TH | 2016 | 230 | 2009-2012 | CSS,OS | Asia | NR | 197/33 | NR | 7 |
| Lim | 2016 | 98 | 2005-2010 | OS | Asia | 69.5 | 82/16 | 34.3 | 7 |
| Liu | 2016 | 296 | 2000-2013 | CSS | Asia | 61.71 | 250/46 | 72 | 7 |
| Ojerholm | 2016 | 230 | 1987-1998 | OS | North America | NR | 185/45 | 223.2 | 7 |
| Zargar | 2016 | 873 | 2000-2013 | OS | International | 63 | 687/196 | 17 | 6 |
| Zargar-  Shoshtari | 2016 | 304 | 2000-2013 | OS | International | 64 | 221/83 | 13 | 6 |
| Anan | 2017 | 532 | 1996-2017 | OS | Asia | NR | 419/113 | NR | 7 |
| Chappidi | 2017 | 169 | 2005-2014 | OS | North America | NR | 145/24 | 18 | 6 |
| Crozier | 2017 | 220 | 2005-2014 | CSS | Asia | 69.5 | 177/43 | NR | 6 |
| D'andrea | 2017 | 448 | 1988-2003 | CSS,OS | North America | 65.2 | 373/75 | 170.4 | 7 |
| Maruf | 2017 | 27451 | 1988-2013 | OS | North America | NR | 20184/7267 | 28 | 8 |
| Matsumoto | 2017 | 594 | 1990-2013 | CSS,OS | Asia | 67 | 482/112 | 48 | 7 |
| Pichler | 2017 | 259 | 2000-2016 | CSS,OS | Europe | 69 | 212/47 | NR | 6 |
| Siemens | 2017 | 2593 | 2000-2008 | CSS,OS | North America | NR | 1949/644 | NR | 6 |
| Soria | 2017 | 424 | 2000-2011 | CSS,OS | Europe | 65.7 | 352/72 | 129 | 7 |
| Vetterlein | 2017 | 611 | 2011 | CSS,OS | Europe | 68.1 | 491/120 | 26 | 7 |
| Xu | 2017 | 162 | 2011-2015 | CSS,OS | Asia | 67 | 118/44 | NR | 7 |
| Zargar | 2017 | 345 | 2000-2015 | OS | International | 62 | 266/79 | 27.8 | 7 |
| Hermans TJN | 2018 | 5517 | 1995-2013 | OS | Europe | NR | 4208/1309 | 110.4 | 7 |
| Martini T | 2018 | 108 | 2008-2011 | CSS | Europe | 72 | 86/22 | 23.2 | 6 |
| Murakami | 2018 | 286 | 1990-2015 | CSS | Asia | 68 | 223/63 | 30.9 | 7 |
| Pietzak | 2018 | 245 | 2001-2015 | CSS,OS | North America | 65 | 181/64 | 48 | 7 |
| Batista | 2019 | 256 | 1994-2013 | OS | North America | 70 | 208/48 | NR | 6 |
| Bi | 2019 | 152 | 2012-2016 | OS | Asia | 69 | 125/27 | 17.4 | 6 |
| Froehner | 2019 | 1268 | 1993-2016 | OS | Europe | 70 | 999/269 | 68.4 | 7 |
| Ha | 2019 | 118 | 2008-2013 | CSS,OS | Asia | 69 | 98/20 | 34.1 | 7 |
| Jin | 2019 | 173 | 2010-2013 | OS | Asia | 61.4 | 152/21 | NR | 6 |
| Marks | 2019 | 517 | 1996-2011 | CSS,OS | Europe | 67 | 400/117 | 45 | 7 |
| Martini A | 2019 | 189 | 2006-2011 | OS | North America | 63 | 146/43 | NR | 6 |
| Turker | 2019 | 236 | 1985-1997 | OS | Europe | NR | 194/42 | NR | 6 |
| Fallah | 2020 | 109 | 2015-2018 | OS | North America | 67 | 94/15 | 18.7 | 6 |
| Volz | 2020 | 118 | 2004-2019 | OS | Europe | 68.9 | 32/86 | 12 | 6 |
| Abbreviations: CSS; cancer-specific survival, F; female, M; male, NR; not reported, OS; overall survival | | | | | | | | | |
