# Supplementary material for: Impact of sex on outcomes after surgery for non-muscle-invasive and muscle-invasive bladder urothelial carcinoma: a systematic review and meta-analysis
Source: World J Urol. 2022 Aug 13;41(4):909–19. doi: 10.1007/s00345-022-04116-x (PMC10159976; doi:10.1007/s00345-022-04116-x)
Supplement: Supplementary file 4 — Supplementary file4 (DOCX 18 KB) [file 345_2022_4116_MOESM4_ESM.docx]

Supplementary Table 2 Study Characteristics (non-muscle invasive bladder cancer)

| Author | Year | N | Recruitment | Outcome | Region | Age | Sex  (M/F) | follow up (M) | NOS |
| --- | --- | --- | --- | --- | --- | --- | --- | --- | --- |
| Hara | 2003 | 97 | 1995-1997 | RFS | Asia | NR | 76/21 | 25.2 | 7 |
| Mitsumori | 2004 | 69 | 1998-2001 | RFS | Asia | 68 | 51/18 | NR | 6 |
| Nonomura | 2006 | 71 | 1995-2001 | RFS | Asia | NR | 50/21 | 21.6 | 6 |
| Sakai | 2006 | 154 | 1988-2004 | RFS | Asia | NR | 131/23 | 81.6 | 7 |
| Herr | 2007 | 215 | 1995-2000 | RFS | North America | NR | 127/88 | 96 | 7 |
| Decobert | 2008 | 111 | 1997-2002 | RFS | North America | NR | 91/20 | 31.2 | 7 |
| Fernandez | 2008 | 1062 | 1990-1999 | RFS | Europe | NR | 945/117 | 69.6 | 7 |
| Gudjonsson | 2008 | 219 | 1997-2004 | RFS | Europe | NR | 155/64 | 43.2 | 6 |
| Jancke | 2008 | 472 | 1992-2001 | RFS | Europe | 72 | 362/110 | 60 | 7 |
| Kikuchi | 2009 | 1710 | 1999-2001 | RFS | Asia | 69.9 | 2600/637 | 24 | 7 |
| Boojian | 2010 | 1021 | 1978-2006 | PFS,RFS | North America | 64 | 756/265 | NR | 6 |
| Lammers | 2011 | 718 | 1998-2004 | RFS | Europe | 66 | 577/141 | 30 | 6 |
| Pellucchi | 2011 | 270 | 2004-2008 | PFS,RFS | Europe | 67.3 | 220/50 | 25.2 | 6 |
| Otto | 2012 | 87 | 2002-2009 | PFS,RFS | Europe | 70 | 72/15 | 31.2 | 7 |
| van Rhijn | 2012 | 129 | 1984-2006 | PFS,RFS | International | NR | 105/24 | 78 | 7 |
| Alvarez | 2013 | 108 | 1989-1996 | CSS,PFS,  RFS | Europe | 65.6 | 100/8 | 76.8 | 6 |
| Chamie | 2013 | 7410 | 1992-2002 | CSS,PFS,  RFS | North America | NR | 5597/1813 | 120 | 7 |
| Kluth | 2013 | 916 | 1996-2007 | CSS,PFS,  RFS | International | 68 | 726/190 | 43.2 | 6 |
| Jancke | 2014 | 768 | 1992-2007 | RFS | Europe | 72 | 591/177 | 56.4 | 7 |
| Rieken | 2014 | 1447 | 1996-2007 | RFS | International | 65 | 1113/323 | 67.2 | 6 |
| Liedberg | 2015 | 5839 | 2004-2007 | PFS,RFS | Europe | NR | 3606/1109 | 60 | 7 |
| Ofude | 2015 | 469 | 2001-2012 | RFS | Asia | 71 | 385/84 | 37.2 | 7 |
| Hurle | 2016 | 74 | 2010-2014 | RFS | Europe | 71 | 58/16 | 27.6 | 7 |
| Abufaraj | 2017 | 827 | NR | CSS,PFS,  RFS | International | 67 | 644/183 | 55 | 7 |
| Soria | 2017 | 1117 | 1996-2007 | CSS,PFS,  RFS | International | 67 | 855/262 | 62.4 | 7 |
| Ucpinar | 2019 | 231 | 2015-2018 | RFS | Europe | 64 | 208/23 | 24 | 5 |
| Yasui | 2019 | 53 | 2008-2015 | RFS | Asia | 74.1 | 48/5 | 10.2 | 6 |
| Mastroianni | 2020 | 521 | 1996-2018 | RFS | Europe | 66 | 422/99 | NR | 6 |
| Abd Elwahab | 2021 | 65 | 2013-2020 | PFS,RFS | Africa | 61.49 | 44/21 | NR | 5 |
| Blindheim | 2021 | 1130 | 2008-2012 | CSS | Europe | 75 | 869/261 | NR | 6 |
| van Rhijn | 2021 | 5145 | 1990-2019 | PFS,RFS | International | 68 | 4125/1020 | 46.8 | 7 |
| Abbreviations: CSS; cancer-specific survival, F; female, M; male, NR; not reported, PFS; progression-free survival, RFS; recurrence-free survival | | | | | | | | | |
