# Supplementary material for: Impact of sex on outcomes after surgery for non-muscle-invasive and muscle-invasive bladder urothelial carcinoma: a systematic review and meta-analysis
Source: World J Urol. 2022 Aug 13;41(4):909–19. doi: 10.1007/s00345-022-04116-x (PMC10159976; doi:10.1007/s00345-022-04116-x)
Supplement: Supplementary file 5 — Supplementary file5 (DOCX 19 KB) [file 345_2022_4116_MOESM5_ESM.docx]

Reference list

Non-muscle invasive bladder carcinoma

1. Hara I, Miyake H, Takechi Y, Eto H, Gotoh A, Fujisawa M, Okada H, Arakawa S, Kamidono S, Kobe Urogenital Tumor Study G (2003) Clinical outcome of conservative therapy for stage T1, grade 3 transitional cell carcinoma of the bladder. International Journal of Urology 10 (1):19-24. doi:10.1046/j.1442-2042.2003.00559.x

2. Mitsumori K, Tsuchiya N, Habuchi T, Li Z, Akao T, Ohyama C, Sato K, Kato T (2004) Early and large-dose intravesical instillation of epirubicin to prevent superficial bladder carcinoma recurrence after transurethral resection. BJU Int 94 (3):317-321. doi:10.1111/j.1464-410X.2004.04884.x

3. Nonomura N, Nakai Y, Nakayama M, Inoue H, Nishimura K, Hatanaka E, Arima R, Kishimoto T, Miki T, Kuroda H, Okuyama A (2006) The expression of thymidine phosphorylase is a prognostic predictor for the intravesical recurrence of superficial bladder cancer. Int J Clin Oncol 11 (4):297-302. doi:10.1007/s10147-006-0562-9

4. Sakai I, Miyake H, Harada KI, Hara I, Inoue TA, Fujisawa M (2006) Analysis of factors predicting intravesical recurrence of superficial transitional cell carcinoma of the bladder without concomitant carcinoma in situ. International Journal of Urology 13 (11):1389-1392. doi:10.1111/j.1442-2042.2006.01562.x

5. Herr HW, Donat SM, Reuter VE (2007) Management of low grade papillary bladder tumors. The Journal of urology 178 (4 Pt 1):1201-1205; discussion 1205. doi:10.1016/j.juro.2007.05.148

6. Decobert M, LaRue H, Harel F, Meyer F, Fradet Y, Lacombe L (2008) Maintenance bacillus Calmette-Guérin in high-risk nonmuscle-invasive bladder cancer: how much is enough? Cancer 113 (4):710-716. doi:10.1002/cncr.23627

7. Fernandez-Gomez J, Solsona E, Unda M, Martinez-Piñeiro L, Gonzalez M, Hernandez R, Madero R, Ojea A, Pertusa C, Rodriguez-Molina J, Camacho JE, Isorna S, Rabadan M, Astobieta A, Montesinos M, Muntañola P, Gimeno A, Blas M, Martinez-Piñeiro JA (2008) Prognostic factors in patients with non-muscle-invasive bladder cancer treated with bacillus Calmette-Guérin: multivariate analysis of data from four randomized CUETO trials. Eur Urol 53 (5):992-1001. doi:10.1016/j.eururo.2007.10.006

8. Gudjónsson S, Adell L, Merdasa F, Olsson R, Larsson B, Davidsson T, Richthoff J, Hagberg G, Grabe M, Bendahl PO, Månsson W, Liedberg F (2009) Should all patients with non-muscle-invasive bladder cancer receive early intravesical chemotherapy after transurethral resection? The results of a prospective randomised multicentre study. Eur Urol 55 (4):773-780. doi:10.1016/j.eururo.2009.01.006

9. Jancke G, Damm O, Rosell J, Jahnson S (2008) Risk factors for local recurrence in patients with pTa/pT1 urinary bladder cancer. Scand J Urol Nephrol 42 (5):417-421. doi:10.1080/00365590802016302

10. Kikuchi E, Fujimoto H, Mizutani Y, Okajima E, Koga H, Hinotsu S, Shinohara N, Oya M, Miki T, Canc Registration C, Japanese Urol A (2009) Clinical outcome of tumor recurrence for Ta, T1 non-muscle invasive bladder cancer from the data on registered bladder cancer patients in Japan: 1999-2001 report from the Japanese Urological Association. International Journal of Urology 16 (3):279-286. doi:10.1111/j.1442-2042.2008.02235.x

11. Boorjian SA, Zhu F, Herr HW (2010) The effect of gender on response to bacillus Calmette-Guérin therapy for patients with non-muscle-invasive urothelial carcinoma of the bladder. BJU Int 106 (3):357-361. doi:10.1111/j.1464-410X.2009.09137.x

12. Lammers RJM, Witjes WPJ, Hendricksen K, Caris CTM, Janzing-Pastors MHC, Witjes JA (2011) Smoking Status Is a Risk Factor for Recurrence After Transurethral Resection of Non-Muscle-Invasive Bladder Cancer. European Urology 60 (4):713-720. doi:10.1016/j.eururo.2011.07.010

13. Otto W, Rubenwolf PC, Burger M, Fritsche HM, Rößler W, May M, Hartmann A, Hofstädter F, Wieland WF, Denzinger S (2012) Loss of aquaporin 3 protein expression constitutes an independent prognostic factor for progression-free survival: an immunohistochemical study on stage pT1 urothelial bladder cancer. BMC Cancer 12:459. doi:10.1186/1471-2407-12-459

14. Pellucchi F, Freschi M, Ibrahim B, Rocchini L, Maccagnano C, Briganti A, Rigatti P, Montorsi F, Colombo R (2011) Clinical reliability of the 2004 WHO histological classification system compared with the 1973 WHO system for Ta primary bladder tumors. The Journal of urology 186 (6):2194-2199. doi:10.1016/j.juro.2011.07.070

15. Van Rhijn BWG, Van Der Kwast TH, Alkhateeb SS, Fleshner NE, Van Leenders GJLH, Bostrom PJ, Van Der Aa MNM, Kakiashvili DM, Bangma CH, Jewett MAS, Zlotta AR (2012) A new and highly prognostic system to discern T1 bladder cancer substage. European Urology 61 (2):378-384. doi:10.1016/j.eururo.2011.10.026

16. Alvarez-Mugica M, Fernandez-Gomez JM, Cebrian V, Fresno F, Escaf S, Sanchez-Carbayo M (2013) Polyamine-modulated Factor-1 Methylation Predicts Bacillus Calmette-Guerin Response in Patients with High-grade Non-muscle-invasive Bladder Carcinoma. European Urology 63 (2):364-370. doi:10.1016/j.eururo.2012.05.050

17. Chamie K, Litwin MS, Bassett JC, Daskivich TJ, Lai J, Hanley JM, Konety BR, Saigal CS (2013) Recurrence of high-risk bladder cancer: a population-based analysis. Cancer 119 (17):3219-3227. doi:10.1002/cncr.28147

18. Kluth LA, Fajkovic H, Xylinas E, Crivelli JJ, Passoni N, Rouprêt M, Becker A, Comploj E, Pycha A, Holmang S, Gupta A, Lotan Y, Karakiewicz PI, Gontero P, Chun FKH, Fisch M, Scherr DS, Shariat SF (2013) Female gender is associated with higher risk of disease recurrence in patients with primary T1 high-grade urothelial carcinoma of the bladder. World Journal of Urology 31 (5):1029-1036. doi:10.1007/s00345-012-0996-9

19. Jancke G, Rosell J, Jahnson S (2014) Impact of surgical experience on recurrence and progression after transurethral resection of bladder tumour in non-muscle-invasive bladder cancer. Scand J Urol 48 (3):276-283. doi:10.3109/21681805.2013.864327

20. Rieken M, Xylinas E, Kluth L, Crivelli JJ, Chrystal J, Faison T, Lotan Y, Karakiewicz PI, Holmäng S, Babjuk M, Fajkovic H, Seitz C, Klatte T, Pycha A, Bachmann A, Scherr DS, Shariat SF (2014) Long-term cancer-specific outcomes of TaG1 urothelial carcinoma of the bladder. Eur Urol 65 (1):201-209. doi:10.1016/j.eururo.2013.08.034

21. Liedberg F, Hagberg O, Holmäng S, Hosseini Aliabad A, Jancke G, Ljungberg B, Malmström PU, Åberg H, Jahnson S (2015) Local recurrence and progression of non-muscle-invasive bladder cancer in Sweden: a population-based follow-up study. Scand J Urol 49 (4):290-295. doi:10.3109/21681805.2014.1000963

22. Ofude M, Kitagawa Y, Yaegashi H, Izumi K, Ueno S, Kadono Y, Konaka H, Mizokami A, Namiki M (2015) Selection of adjuvant intravesical therapies using the European Organization for Research and Treatment of Cancer scoring system in patients at intermediate risk of non-muscle-invasive bladder cancer. J Cancer Res Clin Oncol 141 (1):161-168. doi:10.1007/s00432-014-1795-z

23. Hurle R, Lazzeri M, Colombo P, Buffi NM, Morenghi E, Peschechera R, Castaldo L, Pasini L, Casale P, Seveso M, Zandegiacomo S, Taverna G, Benetti A, Lughezzani G, Fiorini G, Guazzoni G (2016) "En Bloc" Resection of Nonmuscle Invasive Bladder Cancer: A Prospective Single-center Study. Urology 90:126-130. doi:10.1016/j.urology.2016.01.004

24. Abufaraj M, Shariat SF, Haitel A, Moschini M, Foerster B, Chłosta P, Gust K, Babjuk M, Briganti A, Karakiewicz PI, Albrecht W (2017) Prognostic role of N-cadherin expression in patients with non–muscle-invasive bladder cancer. Urologic Oncology: Seminars and Original Investigations 35 (5):264-271. doi:10.1016/j.urolonc.2017.01.012

25. Soria F, Moschini M, Abufaraj M, Wirth GJ, Foerster B, Gust KM, Özsoy M, Briganti A, Gontero P, Mathieu R, Rouprêt M, Karakiewicz PI, Shariat SF (2017) Preoperative anemia is associated with disease recurrence and progression in patients with non-muscle-invasive bladder cancer. Urologic oncology 35 (3):113.e119-113.e114. doi:10.1016/j.urolonc.2016.10.021

26. Ucpinar B, Erbin A, Ayranci A, Caglar U, Alis D, Basal S, Sarilar O, Akbulut MF (2019) Prediction of recurrence in non-muscle invasive bladder cancer patients. Do patient characteristics matter? Journal of BUON : official journal of the Balkan Union of Oncology 24 (4):1659-1665

27. Yasui M, Kawahara T, Izumi K, Yao M, Ishiguro Y, Ishiguro H, Uemura H, Miyoshi Y (2019) Androgen receptor mRNA expression is a predictor for recurrence-free survival in non-muscle invasive bladder cancer. BMC Cancer 19 (1):331. doi:10.1186/s12885-019-5512-9

28. Mastroianni R, Brassetti A, Krajewsky W, Zdrojowy R, Salhi YA, Anceschi U, Bove AM, Carbone A, De Nunzio C, Fuschi A, Ferriero M, Nacchia A, Pastore AL, Tema G, Tuderti G, Gallucci M, Simone G (2020) Assessing the Impact of the Absence of Detrusor Muscle in Ta Low-grade Urothelial Carcinoma of the Bladder on Recurrence-free Survival. European Urology Focus. doi:10.1016/j.euf.2020.08.007

29. Abd Elwahab KM, Desky EAE, Eldery MS, Mohammad FF, Seleem MM, El-Babouly IM (2021) Apparent Diffusion Coefficient Value can Predict Poor Bacillus Calmette–Guérin Responders in T1HG/NMIBC: Prospective Cohort Study. Clinical Genitourinary Cancer 19 (4):e248-e254. doi:10.1016/j.clgc.2021.03.001

30. Blindheim AJ, Fosså SD, Babigumira R, Andreassen BK (2021) The use of reTURB in T1 bladder cancer: a Norwegian population-based study. Scand J Urol 55 (4):268-274. doi:10.1080/21681805.2021.1917652

31. van Rhijn BWG, Hentschel AE, Bründl J, Compérat EM, Hernández V, Čapoun O, Bruins HM, Cohen D, Rouprêt M, Shariat SF, Mostafid AH, Zigeuner R, Dominguez-Escrig JL, Burger M, Soukup V, Gontero P, Palou J, van der Kwast TH, Babjuk M, Sylvester RJ (2021) Prognostic Value of the WHO1973 and WHO2004/2016 Classification Systems for Grade in Primary Ta/T1 Non-muscle-invasive Bladder Cancer: A Multicenter European Association of Urology Non-muscle-invasive Bladder Cancer Guidelines Panel Study. Eur Urol Oncol 4 (2):182-191. doi:10.1016/j.euo.2020.12.002
