# Supplementary material for: Impact of sex on outcomes after surgery for non-muscle-invasive and muscle-invasive bladder urothelial carcinoma: a systematic review and meta-analysis
Source: World J Urol. 2022 Aug 13;41(4):909–19. doi: 10.1007/s00345-022-04116-x (PMC10159976; doi:10.1007/s00345-022-04116-x)
Supplement: Supplementary file 6 — Supplementary file6 (DOCX 24 KB) [file 345_2022_4116_MOESM6_ESM.docx]

Reference list

Muscle invasive bladder carcinoma

1. Tilki D, Svatek RS, Novara G, Seitz M, Godoy G, Karakiewicz PI, Kassouf W, Fradet Y, Fritsche HM, Sonpavde G, Izawa JI, Ficarra V, Lerner SP, Schoenberg M, Stief CG, Dinney CP, Skinner E, Lotan Y, Sagalowsky AI, Reich O, Shariat SF (2010) Stage pT0 at radical cystectomy confers improved survival: an international study of 4,430 patients. The Journal of urology 184 (3):888-894. doi:10.1016/j.juro.2010.04.081

2. Boström PJ, Alkhateeb S, Trottier G, Athanasopoulos PZ, Mirtti T, Kortekangas H, Laato M, van Rhijn B, van der Kwast T, Fleshner NE, Jewett MA, Finelli A, Zlotta AR (2012) Sex differences in bladder cancer outcomes among smokers with advanced bladder cancer. BJU Int 109 (1):70-76. doi:10.1111/j.1464-410X.2011.10371.x

3. Jensen JB, Ulhoi BP, Jensen KME (2012) Evaluation of different lymph node (LN) variables as prognostic markers in patients undergoing radical cystectomy and extended LN dissection to the level of the inferior mesenteric artery. Bju International 109 (3):388-393. doi:10.1111/j.1464-410X.2011.10369.x

4. Gregg JR, Cookson MS, Phillips S, Salem S, Chang SS, Clark PE, Davis R, Stimson CJ, Jr., Aghazadeh M, Smith JA, Jr., Barocas DA (2011) Effect of preoperative nutritional deficiency on mortality after radical cystectomy for bladder cancer. The Journal of urology 185 (1):90-96. doi:10.1016/j.juro.2010.09.021

5. Otto W, May M, Fritsche HM, Dragun D, Aziz A, Gierth M, Trojan L, Herrmann E, Moritz R, Ellinger J, Tilki D, Buchner A, Höfner T, Brookman-May S, Nuhn P, Gilfrich C, Roigas J, Zacharias M, Denzinger S, Hohenfellner M, Haferkamp A, Müller SC, Kocot A, Riedmiller H, Wieland WF, Stief CG, Bastian PJ, Burger M (2012) Analysis of sex differences in cancer-specific survival and perioperative mortality following radical cystectomy: results of a large German multicenter study of nearly 2500 patients with urothelial carcinoma of the bladder. Gend Med 9 (6):481-489. doi:10.1016/j.genm.2012.11.001

6. Chromecki TF, Cha EK, Fajkovic H, Rink M, Ehdaie B, Svatek RS, Karakiewicz PI, Lotan Y, Tilki D, Bastian PJ, Daneshmand S, Kassouf W, Durand M, Novara G, Fritsche HM, Burger M, Izawa JI, Brisuda A, Babjuk M, Pummer K, Shariat SF (2013) Obesity is associated with worse oncological outcomes in patients treated with radical cystectomy. BJU Int 111 (2):249-255. doi:10.1111/j.1464-410X.2012.11322.x

7. da Silva RD, Xylinas E, Kluth L, Crivelli JJ, Chrystal J, Chade D, Guglielmetti GB, Pycha A, Lotan Y, Karakiewicz PI, Sun M, Fajkovic H, Zerbib M, Scherr DS, Shariat SF (2013) Impact of statin use on oncologic outcomes in patients with urothelial carcinoma of the bladder treated with radical cystectomy. The Journal of urology 190 (2):487-492. doi:10.1016/j.juro.2013.02.003

8. Fajkovic H, Cha EK, Jeldres C, Robinson BD, Rink M, Xylinas E, Chromecki TF, Breinl E, Svatek RS, Donner G, Tagawa ST, Tilki D, Bastian PJ, Karakiewicz PI, Volkmer BG, Novara G, Joual A, Faison T, Sonpavde G, Daneshmand S, Lotan Y, Scherr DS, Shariat SF (2013) Extranodal extension is a powerful prognostic factor in bladder cancer patients with lymph node metastasis. Eur Urol 64 (5):837-845. doi:10.1016/j.eururo.2012.07.026

9. Fritsche HM, May M, Denzinger S, Otto W, Siegert S, Giedl C, Giedl J, Eder F, Agaimy A, Novotny V, Wirth M, Stief C, Brookman-May S, Hofstädter F, Gierth M, Aziz A, Kocot A, Riedmiller H, Bastian PJ, Toma M, Wieland WF, Hartmann A, Burger M (2013) Prognostic value of perinodal lymphovascular invasion following radical cystectomy for lymph node-positive urothelial carcinoma. Eur Urol 63 (4):739-744. doi:10.1016/j.eururo.2012.09.053

10. May M, Bastian PJ, Brookman-May S, Fritsche HM, Tilki D, Otto W, Bolenz C, Gilfrich C, Trojan L, Herrmann E, Moritz R, Tiemann A, Müller SC, Ellinger J, Buchner A, Stief CG, Wieland WF, Höfner T, Hohenfellner M, Haferkamp A, Roigas J, Zacharias M, Nuhn P, Burger M (2013) Gender-specific differences in cancer-specific survival after radical cystectomy for patients with urothelial carcinoma of the urinary bladder in pathologic tumor stage T4a. Urologic oncology 31 (7):1141-1147. doi:10.1016/j.urolonc.2011.09.011

11. Morikawa T, Kawai T, Abe H, Kume H, Homma Y, Fukayama M (2013) UBE2C is a marker of unfavorable prognosis in bladder cancer after radical cystectomy. International journal of clinical and experimental pathology 6 (7):1367-1374

12. Abel EJ, Linder BJ, Bauman TM, Bauer RM, Thompson RH, Thapa P, Devon ON, Tarrell RF, Frank I, Jarrard DF, Downs TM, Boorjian SA (2014) Perioperative blood transfusion and radical cystectomy: does timing of transfusion affect bladder cancer mortality? Eur Urol 66 (6):1139-1147. doi:10.1016/j.eururo.2014.08.051

13. Breyer J, Denzinger S, Otto W, Bründl J, Gierth M, Fritsche HM, Rößler W, Wieland WF, Giedl C, Hofstädter F, Rubenwolf P, Burger M, Aziz A (2014) Outcome of patients with pathological tumor stage T3 urothelial carcinoma of the bladder following radical cystectomy in a single-center series with 116 patients. Urol Int 93 (3):311-319. doi:10.1159/000360483

14. Hermanns T, Bhindi B, Wei Y, Yu J, Noon AP, Richard PO, Bhatt JR, Almatar A, Jewett MA, Fleshner NE, Zlotta AR, Templeton AJ, Kulkarni GS (2014) Pre-treatment neutrophil-to-lymphocyte ratio as predictor of adverse outcomes in patients undergoing radical cystectomy for urothelial carcinoma of the bladder. Br J Cancer 111 (3):444-451. doi:10.1038/bjc.2014.305

15. Kluth LA, Rieken M, Xylinas E, Kent M, Rink M, Rouprêt M, Sharifi N, Jamzadeh A, Kassouf W, Kaushik D, Boorjian SA, Roghmann F, Noldus J, Masson-Lecomte A, Vordos D, Ikeda M, Matsumoto K, Hagiwara M, Kikuchi E, Fradet Y, Izawa J, Rendon R, Fairey A, Lotan Y, Bachmann A, Zerbib M, Fisch M, Scherr DS, Vickers A, Shariat SF (2014) Gender-specific differences in clinicopathologic outcomes following radical cystectomy: an international multi-institutional study of more than 8000 patients. Eur Urol 66 (5):913-919. doi:10.1016/j.eururo.2013.11.040

16. May M, Burger M, Brookman-May S, Stief CG, Fritsche HM, Roigas J, Zacharias M, Bader M, Mandel P, Gilfrich C, Seitz M, Tilki D (2014) EORTC progression score identifies patients at high risk of cancer-specific mortality after radical cystectomy for secondary muscle-invasive bladder cancer. Clin Genitourin Cancer 12 (4):278-286. doi:10.1016/j.clgc.2013.11.014

17. Messer JC, Shariat SF, Dinney CP, Novara G, Fradet Y, Kassouf W, Karakiewicz PI, Fritsche HM, Izawa JI, Lotan Y, Skinner EC, Tilki D, Ficarra V, Volkmer BG, Isbarn H, Wei CM, Lerner SP, Curiel TJ, Kamat AM, Svatek RS (2014) Female Gender Is Associated With a Worse Survival After Radical Cystectomy for Urothelial Carcinoma of the Bladder: A Competing Risk Analysis. Urology 83 (4):863-867. doi:10.1016/j.urology.2013.10.060

18. Kwon T, Jeong IG, You D, Han KS, Hong S, Hong B, Hong JH, Ahn H, Kim CS (2014) Obesity and prognosis in muscle-invasive bladder cancer: the continuing controversy. International journal of urology : official journal of the Japanese Urological Association 21 (11):1106-1112. doi:10.1111/iju.12530

19. Abdi H, Pourmalek F, Gleave ME, So AI, Black PC (2016) Balancing risk and benefit of extended pelvic lymph node dissection in patients undergoing radical cystectomy. World J Urol 34 (1):41-48. doi:10.1007/s00345-015-1734-x

20. Aziz A, Shariat SF, Roghmann F, Brookman-May S, Stief CG, Rink M, Chun FK, Fisch M, Novotny V, Froehner M, Wirth MP, Schnabel MJ, Fritsche HM, Burger M, Pycha A, Brisuda A, Babjuk M, Vallo S, Haferkamp A, Roigas J, Noldus J, Stredele R, Volkmer B, Bastian PJ, Xylinas E, May M (2016) Prediction of cancer-specific survival after radical cystectomy in pT4a urothelial carcinoma of the bladder: development of a tool for clinical decision-making. BJU Int 117 (2):272-279. doi:10.1111/bju.12984

21. Gaisa NT, Wilms H, Wild PJ, Jakse G, Heidenreich A, Knuechel R (2015) In cystectomy specimens with bladder cancer whole organ embedding increases the detection rate of histopathological parameters, but not of those with prognostic significance. Virchows Archiv : an international journal of pathology 466 (4):423-432. doi:10.1007/s00428-015-1726-7

22. Kim HS, Piao S, Moon KC, Jeong CW, Kwak C, Kim HH, Ku JH (2015) Adjuvant Chemotherapy Correlates with Improved Survival after Radical Cystectomy in Patients with pT3b (Macroscopic Perivesical Tissue Invasion) Bladder Cancer. J Cancer 6 (8):750-758. doi:10.7150/jca.12259

23. Moschini M, Dell' Oglio P, Capogrosso P, Cucchiara V, Luzzago S, Gandaglia G, Zattoni F, Briganti A, Damiano R, Montorsi F, Salonia A, Colombo R (2015) Effect of Allogeneic Intraoperative Blood Transfusion on Survival in Patients Treated With Radical Cystectomy for Nonmetastatic Bladder Cancer: Results From a Single High-Volume Institution. Clin Genitourin Cancer 13 (6):562-567. doi:10.1016/j.clgc.2015.04.009

24. Patel MI, Bang A, Gillatt D, Smith DP (2015) Contemporary radical cystectomy outcomes in patients with invasive bladder cancer: a population-based study. BJU Int 116 Suppl 3:18-25. doi:10.1111/bju.13152

25. Raza SJ, Wilson T, Peabody JO, Wiklund P, Scherr DS, Al-Daghmin A, Dibaj S, Khan MS, Dasgupta P, Mottrie A, Menon M, Yuh B, Richstone L, Saar M, Stoeckle M, Hosseini A, Kaouk J, Mohler JL, Rha KH, Wilding G, Guru KA (2015) Long-term oncologic outcomes following robot-assisted radical cystectomy: results from the International Robotic Cystectomy Consortium. Eur Urol 68 (4):721-728. doi:10.1016/j.eururo.2015.04.021

26. Satkunasivam R, Hu B, Metcalfe C, Ghodoussipour SB, Aron M, Cai J, Miranda G, Gill I, Daneshmand S (2016) Utility and significance of ureteric frozen section analysis during radical cystectomy. BJU Int 117 (3):463-468. doi:10.1111/bju.13081

27. Tabata M, Ikeda M, Urakami S, Takahashi S, Sakaguchi K, Kurosawa K, Okaneya T (2015) Impact of adjuvant chemotherapy on patients with pathological Stage T3b and/or lymph node metastatic bladder cancer after radical cystectomy. Jpn J Clin Oncol 45 (10):963-967. doi:10.1093/jjco/hyv098

28. Dabi Y, Rouscoff Y, Anract J, Delongchamps NB, Sibony M, Saighi D, Zerbib M, Peyraumore M, Xylinas E (2017) Impact of body mass index on the oncological outcomes of patients treated with radical cystectomy for muscle-invasive bladder cancer. World J Urol 35 (2):229-235. doi:10.1007/s00345-016-1852-0

29. D'Andrea D, Moschini M, Gust KM, Abufaraj M, Özsoy M, Mathieu R, Soria F, Briganti A, Rouprêt M, Karakiewicz PI, Shariat SF (2017) Lymphocyte-to-monocyte ratio and neutrophil-to-lymphocyte ratio as biomarkers for predicting lymph node metastasis and survival in patients treated with radical cystectomy. J Surg Oncol 115 (4):455-461. doi:10.1002/jso.24521

30. Gershman B, Moreira DM, Tollefson MK, Frank I, Cheville JC, Thapa P, Tarrell RF, Thompson RH, Boorjian SA (2016) The association of ABO blood type with disease recurrence and mortality among patients with urothelial carcinoma of the bladder undergoing radical cystectomy. Urologic oncology 34 (1):4.e1-9. doi:10.1016/j.urolonc.2015.07.023

31. Kaimakliotis HZ, Monn MF, Cho JS, Pedrosa JA, Hahn NM, Albany C, Gellhaus PT, Cary KC, Masterson TA, Foster RS, Bihrle R, Cheng L, Koch MO (2016) Neoadjuvant chemotherapy in urothelial bladder cancer: impact of regimen and variant histology. Future oncology (London, England) 12 (15):1795-1804. doi:10.2217/fon-2016-0056

32. Lim S, Koh MJ, Jeong HJ, Cho NH, Choi YD, Cho do Y, Lee HY, Rha SY (2016) Fibroblast Growth Factor Receptor 1 Overexpression Is Associated with Poor Survival in Patients with Resected Muscle Invasive Urothelial Carcinoma. Yonsei Med J 57 (4):831-839. doi:10.3349/ymj.2016.57.4.831

33. Kim TH, Sung HH, Jeon HG, Seo SI, Jeon SS, Lee HM, Choi HY, Jeong BC (2016) Oncological Outcomes in Patients Treated with Radical Cystectomy for Bladder Cancer: Comparison Between Open, Laparoscopic, and Robot-Assisted Approaches. J Endourol 30 (7):783-791. doi:10.1089/end.2015.0652

34. Liu J, Dai Y, Zhou F, Long Z, Li Y, Liu B, Xie D, Tang J, Tan J, Yao K, Zhang Y, Tang Y, He L (2016) The prognostic role of preoperative serum albumin/globulin ratio in patients with bladder urothelial carcinoma undergoing radical cystectomy. Urologic oncology 34 (11):484.e481-484.e488. doi:10.1016/j.urolonc.2016.05.024

35. Ojerholm E, Smith A, Hwang WT, Baumann BC, Tucker KN, Lerner SP, Mamtani R, Boursi B, Christodouleas JP (2017) Neutrophil-to-lymphocyte ratio as a bladder cancer biomarker: Assessing prognostic and predictive value in SWOG 8710. Cancer 123 (5):794-801. doi:10.1002/cncr.30422

36. Zargar H, Zargar-Shoshtari K, Lotan Y, Shah JB, van Rhijn BW, Daneshmand S, Spiess PE, Black P (2016) Final Pathological Stage after Neoadjuvant Chemotherapy and Radical Cystectomy for Bladder Cancer-Does pT0 Predict Better Survival than pTa/Tis/T1? The Journal of urology 195 (4 Pt 1):886-893. doi:10.1016/j.juro.2015.10.133

37. Zargar-Shoshtari K, Zargar H, Lotan Y, Shah JB, van Rhijn BW, Daneshmand S, Spiess PE, Black PC (2016) A Multi-Institutional Analysis of Outcomes of Patients with Clinically Node Positive Urothelial Bladder Cancer Treated with Induction Chemotherapy and Radical Cystectomy. The Journal of urology 195 (1):53-59. doi:10.1016/j.juro.2015.07.085

38. Anan G, Hatakeyama S, Fujita N, Iwamura H, Tanaka T, Yamamoto H, Tobisawa Y, Yoneyama T, Yoneyama T, Hashimoto Y, Koie T, Ito H, Yoshikawa K, Kawaguchi T, Sato M, Ohyama C (2017) Trends in neoadjuvant chemotherapy use and oncological outcomes for muscle-invasive bladder cancer in Japan: a multicenter study. Oncotarget 8 (49):86130-86142. doi:10.18632/oncotarget.20991

39. Chappidi MR, Kates M, Brant A, Baras AS, Netto GJ, Pierorazio PM, Hahn NM, Bivalacqua TJ (2017) Assessing Cancer Progression and Stable Disease After Neoadjuvant Chemotherapy for Organ-confined Muscle-invasive Bladder Cancer. Urology 102:148-158. doi:10.1016/j.urology.2016.10.064

40. Crozier J, Papa N, Perera M, Stewart M, Goad J, Sengupta S, Bolton D, Lawrentschuk N (2017) Lymph node yield in node-negative patients predicts cancer specific survival following radical cystectomy for transitional cell carcinoma. Investig Clin Urol 58 (6):416-422. doi:10.4111/icu.2017.58.6.416

41. Maruf M, Sidana A, Purnell S, Jain AL, Brancato SJ, Agarwal PK (2018) Lymph node dissection during radical cystectomy following prior radiation therapy: results from the SEER database. Int Urol Nephrol 50 (2):257-262. doi:10.1007/s11255-017-1751-3

42. Matsumoto A, Nakagawa T, Kanatani A, Ikeda M, Kawai T, Miyakawa J, Taguchi S, Naito A, Otsuka M, Nakanishi Y, Suzuki M, Koga F, Nagase Y, Kondo Y, Okaneya T, Tanaka Y, Miyazaki H, Fujimura T, Fukuhara H, Kume H, Igawa Y, Homma Y (2018) Preoperative chronic kidney disease is predictive of oncological outcome of radical cystectomy for bladder cancer. World J Urol 36 (2):249-256. doi:10.1007/s00345-017-2141-2

43. Pichler R, Fritz J, Heidegger I, Oberaigner W, Horninger W, Hochleitner M (2017) Gender-related Outcome in Bladder Cancer Patients undergoing Radical Cystectomy. J Cancer 8 (17):3567-3574. doi:10.7150/jca.21130

44. Siemens DR, Jaeger MT, Wei XJ, Vera-Badillo F, Booth CM (2017) Peri-operative allogeneic blood transfusion and outcomes after radical cystectomy: a population-based study. World Journal of Urology 35 (9):1435-1442. doi:10.1007/s00345-017-2009-5

45. Soria F, Lucca I, Moschini M, Mathieu R, Rouprêt M, Karakiewicz PI, Briganti A, Rink M, Gust KM, Hassler MR, Foerster B, Abufarraj M, Haitel A, Klatte T, Shariat SF (2017) Caveolin-1 as prognostic factor of disease recurrence and survival in patients treated with radical cystectomy for bladder cancer. Urologic oncology 35 (6):356-362. doi:10.1016/j.urolonc.2017.02.009

46. Vetterlein MW, Gild P, Kluth LA, Seisen T, Gierth M, Fritsche HM, Burger M, Protzel C, Hakenberg OW, von Landenberg N, Roghmann F, Noldus J, Nuhn P, Pycha A, Rink M, Chun FK, May M, Fisch M, Aziz A (2018) Peri-operative allogeneic blood transfusion does not adversely affect oncological outcomes after radical cystectomy for urinary bladder cancer: a propensity score-weighted European multicentre study. BJU Int 121 (1):101-110. doi:10.1111/bju.14012

47. Xu K, Lang B, Fu B, Shi T, Wang B, Zhang X (2017) Laparoendoscopic Single-Site Radical Cystectomy vs Conventional Laparoscopic Radical Cystectomy for Patient with Bladder Urothelial Carcinoma: Matched Case-Control Analysis. J Endourol 31 (12):1259-1268. doi:10.1089/end.2017.0525

48. Zargar H, Shah JB, van de Putte EEF, Potvin KR, Zargar-Shoshtari K, van Rhijn BW, Daneshmand S, Holzbeierlein JM, Spiess PE, Winquist E, Horenblas S, Dinney C, Black PC, Kassouf W (2017) Dose dense MVAC prior to radical cystectomy: a real-world experience. World J Urol 35 (11):1729-1736. doi:10.1007/s00345-017-2065-x

49. Hermans TJN, Voskuilen CS, Deelen M, Mertens LS, Horenblas S, Meijer RP, Boormans JL, Aben KK, van der Heijden MS, Pos FJ, de Wit R, Beerepoot LV, Verhoeven RHA, van Rhijn BWG (2019) Superior efficacy of neoadjuvant chemotherapy and radical cystectomy in cT3-4aN0M0 compared to cT2N0M0 bladder cancer. Int J Cancer 144 (6):1453-1459. doi:10.1002/ijc.31833

50. Martini T, Heinkele J, Mayr R, Weis CA, Wezel F, Wahby S, Eckstein M, Schnöller T, Breyer J, Wirtz R, Ritter M, Bolenz C, Erben P (2018) Predictive value of lymphangiogenesis and proliferation markers on mRNA level in urothelial carcinoma of the bladder after radical cystectomy. Urologic oncology 36 (12):530.e519-530.e527. doi:10.1016/j.urolonc.2018.09.003

51. Murakami Y, Matsumoto K, Ikeda M, Utsunomiya T, Hirayama T, Koguchi D, Matsuda D, Okuno N, Taoka Y, Irie A, Iwamura M (2018) Impact of body mass index on the oncological outcomes of patients with upper and lower urinary tract cancers treated with radical surgery: A multi-institutional retrospective study. Asia-Pacific journal of clinical oncology 14 (4):310-317. doi:10.1111/ajco.12848

52. Pietzak EJ, Zabor EC, Bagrodia A, Armenia J, Hu W, Zehir A, Funt S, Audenet F, Barron D, Maamouri N, Li Q, Teo MY, Arcila ME, Berger MF, Schultz N, Dalbagni G, Herr HW, Bajorin DF, Rosenberg JE, Al-Ahmadie H, Bochner BH, Solit DB, Iyer G (2019) Genomic Differences Between "Primary" and "Secondary" Muscle-invasive Bladder Cancer as a Basis for Disparate Outcomes to Cisplatin-based Neoadjuvant Chemotherapy. Eur Urol 75 (2):231-239. doi:10.1016/j.eururo.2018.09.002

53. Bi H, Huang Y, Wang GL, Ma LL, Lu M (2020) Impact of Body Mass Index and Pretreatment Hemoglobin Level on Prognosis Following Radical Cystectomy for Bladder Cancer in Males and Females. Urologia Internationalis 104 (1-2):28-35. doi:10.1159/000500561

54. Batista da Costa J, Gibb EA, Bivalacqua TJ, Liu Y, Oo HZ, Miyamoto DT, Alshalalfa M, Davicioni E, Wright J, Dall'Era MA, Douglas J, Boormans JL, Van der Heijden MS, Wu CL, van Rhijn BWG, Gupta S, Grivas P, Mouw KW, Murugan P, Fazli L, Ra S, Konety BR, Seiler R, Daneshmand S, Mian OY, Efstathiou JA, Lotan Y, Black PC (2019) Molecular Characterization of Neuroendocrine-like Bladder Cancer. Clinical cancer research : an official journal of the American Association for Cancer Research 25 (13):3908-3920. doi:10.1158/1078-0432.ccr-18-3558

55. Froehner M, Muallah D, Koch R, Hübler M, Borkowetz A, Heberling U, Huber J, Wirth MP, Thomas C (2020) Socioeconomic Status-Related Parameters as Predictors of Competing (Non-Bladder Cancer) Mortality after Radical Cystectomy. Urol Int 104 (1-2):62-69. doi:10.1159/000502781

56. Ha YS, Kim SW, Chun SY, Chung JW, Choi SH, Lee JN, Kim BS, Kim HT, Yoo ES, Kwon TG, Kim WT, Kim WJ, Kim TH (2019) Association between De Ritis ratio (aspartate aminotransferase/alanine aminotransferase) and oncological outcomes in bladder cancer patients after radical cystectomy. BMC Urol 19 (1):10. doi:10.1186/s12894-019-0439-7

57. Jin S, Wang B, Zhu Y, Dai W, Xu P, Yang C, Shen Y, Ye D (2019) Log Odds Could Better Predict Survival in Muscle-Invasive Bladder Cancer Patients Compared with pN and Lymph Node Ratio. J Cancer 10 (1):249-256. doi:10.7150/jca.27399

58. Marks P, Gild P, Soave A, Janisch F, Minner S, Engel O, Vetterlein MW, Shariat SF, Sauter G, Dahlem R, Fisch M, Rink M (2019) The impact of variant histological differentiation on extranodal extension and survival in node positive bladder cancer treated with radical cystectomy. Surg Oncol 28:208-213. doi:10.1016/j.suronc.2019.01.008

59. Martini A, Jia R, Ferket BS, Waingankar N, Plimack ER, Crabb SJ, Harshman LC, Yu EY, Powles T, Rosenberg JE, Pal SK, Vaishampayan UN, Necchi A, Wiklund NP, Mehrazin R, Mazumdar M, Sfakianos JP, Galsky MD (2019) Tumor downstaging as an intermediate endpoint to assess the activity of neoadjuvant systemic therapy in patients with muscle-invasive bladder cancer. Cancer 125 (18):3155-3163. doi:10.1002/cncr.32169

60. Turker P, Segersten U, Malmström PU, Hemdan T (2019) Is Bcl-2 a predictive marker of neoadjuvant chemotherapy response in patients with urothelial bladder cancer undergoing radical cystectomy? Scand J Urol 53 (1):45-50. doi:10.1080/21681805.2019.1575467

61. Fallah J, Diaz-Montero CM, Rayman P, Wei W, Finke JH, Kim JS, Pavicic PG, Jr., Lamenza M, Dann P, Company D, Stephenson A, Campbell S, Haber G, Lee B, Mian O, Gilligan T, Garcia JA, Rini B, Ornstein MC, Grivas P (2020) Myeloid-Derived Suppressor Cells in Nonmetastatic Urothelial Carcinoma of Bladder Is Associated With Pathologic Complete Response and Overall Survival. Clin Genitourin Cancer 18 (6):500-508. doi:10.1016/j.clgc.2020.03.004

62. Volz Y, Grimm T, Ormanns S, Eismann L, Pfitzinger PL, Jokisch JF, Schulz G, Casuscelli J, Schlenker B, Karl A, Stief CG, Kretschmer A (2021) Radical cystectomy for locally advanced urothelial carcinoma of the urinary bladder: Health-related quality of life, oncological outcomes and predictors for survival. Urologic oncology 39 (5):299.e215-299.e221. doi:10.1016/j.urolonc.2020.10.076
